# Overnight Fasting Regulates Inhibitory Tone to Cholinergic Neurons of the Dorsomedial Nucleus of the Hypothalamus

**DOI:** 10.1371/journal.pone.0060828

**Published:** 2013-04-09

**Authors:** Florian Groessl, Jae Hoon Jeong, David A. Talmage, Lorna W. Role, Young-Hwan Jo

**Affiliations:** 1 Department of Neurobiology and Behavior, Stony Brook University, Stony Brook, New York, United States of America; 2 Departments of Medicine and Molecular Pharmacology, Albert Einstein College of Medicine of Yeshiva University, Bronx, New York, United States of America; 3 Research Institute of Molecular Pathology (IMP), Vienna Biocenter, Vienna, Vienna, Austria; University of Wuerzburg, Germany

## Abstract

The dorsomedial nucleus of the hypothalamus (DMH) contributes to the regulation of overall energy homeostasis by modulating energy intake as well as energy expenditure. Despite the importance of the DMH in the control of energy balance, DMH-specific genetic markers or neuronal subtypes are poorly defined. Here we demonstrate the presence of cholinergic neurons in the DMH using genetically modified mice that express enhanced green florescent protein (eGFP) selectively in choline acetyltransferase (Chat)-neurons. Overnight food deprivation increases the activity of DMH cholinergic neurons, as shown by induction of fos protein and a significant shift in the baseline resting membrane potential. DMH cholinergic neurons receive both glutamatergic and GABAergic synaptic input, but the activation of these neurons by an overnight fast is due entirely to decreased inhibitory tone. The decreased inhibition is associated with decreased frequency and amplitude of GABAergic synaptic currents in the cholinergic DMH neurons, while glutamatergic synaptic transmission is not altered. As neither the frequency nor amplitude of miniature GABAergic or glutamatergic postsynaptic currents is affected by overnight food deprivation, the fasting-induced decrease in inhibitory tone to cholinergic neurons is dependent on superthreshold activity of GABAergic inputs. This study reveals that cholinergic neurons in the DMH readily sense the availability of nutrients and respond to overnight fasting via decreased GABAergic inhibitory tone. As such, altered synaptic as well as neuronal activity of DMH cholinergic neurons may play a critical role in the regulation of overall energy homeostasis.

## Introduction

Obesity increases the risk of a number of health conditions including cardiovascular disease, type 2 diabetes, and several cancers [1]. While obesity results from prolonged positive energy balance (i.e. energy intake exceeding energy expenditure), the cause of excessive positive energy balance in obesity has not been clearly defined. Key regulatory components reside in the hypothalamus (for reviews see [2]–[4]). Amongst hypothalamic nuclei, the dorsomedial nucleus of the hypothalamus (DMH) is a critical structure for the regulation of a wide range of physiological processes, ranging from reproduction, thermogenesis, stress response, food intake, and circadian rhythms ([5]–[8] and for reviews see [9]–[11]).

Recent studies have demonstrated the existence of various neurotransmitters and signaling proteins that affect and/or are affected with altered food intake in the DMH. These include leptin-responsive GABAergic neurons [8], [12], brain-derived neurotrophic factor (BDNF) [13], neuropeptide Y (NPY) [5], endocannabinoids, and nitric oxide (NO) [14]. Leptin receptor-expressing neurons in the DMH contribute to the regulation of sympathetic brown adipose tissue outputs, implying that these neurons represent a subset of thermoregulatory circuits [8]. Deletion of BDNF or NPY in the DMH induces opposing effects on food intake [5], [13]. Endocannabinoids and NO that are co-released from DMH neurons differentially regulate GABAergic inhibitory tone and fasting reinforces NO-mediated enhancement of GABAergic currents [14]. Although a recent study further identifies genes that are highly expressed in the DMH using microarray analysis [15], little information is available about molecular markers specific for the DMH, which would facilitate the development of mouse models with DMH-specific genetic manipulations.

Central cholinergic circuits, and the consequent activation of both nicotinic and muscarinic receptor-mediated components, appear to play a role in the regulation of ingestive behavior [16]. In particular, activation of CNS nicotinic receptors leads to a reduction in energy intake via modulation of melanocortinergic neurons such as pro-opiomelanocortin (POMC) and agouti-related peptide (AgRP) neurons in the arcuate nucleus [17], [18]. In contrast, mice lacking the M3 muscarinic receptor show a significant decrease in food intake and body weight. Genetic deletion of the M3 receptors is associated with altered expression of AgRP, POMC and melanin-concentrating hormone (MCH) peptides that are expressed in the arcuate and lateral hypothalamus [19]. At least, one study prior to this showed a cluster of cholinergic neurons in the DMH, but the function of these DMH cholinergic neurons was unknown. [20]. DMH neurons send abundant projections to the paraventricular nucleus, preoptic area, arcuate nucleus, and lateral hypothalamus [21]. It is thus plausible that, at least, a subset of DMH neurons are cholinergic and that the DMH cholinergic neurons play a role in overall energy balance via interactions between the DMH and other hypothalamic nuclei, including the arcuate and lateral hypothalamic nuclei.

Using a BAC transgenic mouse model where cholinergic neurons are labeled with the *tau*GFP fusion protein driven by the choline acetyltransferase promoter [22], we first examined whether we could detect cholinergic neurons in the DMH. We then tested whether synaptic activity of the DMH cholinergic neurons was altered by changes in the availability of nutrients. We found that a single, overnight food deprivation increased fos protein in the DMH cholinergic neurons, as compared to control. This was associated with increased baseline resting membrane potential and decreased inhibitory tone onto cholinergic neurons. Thus, our data indicate that cholinergic neurons within the DMH are a good nutrient-sensitive neuronal marker within this area and that these cholinergic neurons may play an essential role in hypothalamic synapses and circuits that regulate overall energy balance.

## Materials and Methods

### Animals

All mouse care and experimental procedures were approved by the Institutional Animal Care Research Advisory Committee of Stony Brook University. The BAC transgenic – Chat eGFP line was the generous gift of Dr. Sukimar Vijayaraghavan [22].

### Slice Preparation

Transverse brain slices were prepared at postnatal age 2 months ±1 week. Animals were anesthetized with a mixture of ketamine and xylazine. After decapitation, the brain was transferred into a sucrose-based solution bubbled with 95% O_2_/5% CO_2_ and maintained at ∼4°C. This solution contained the following (in mM): 248 sucrose, 2 KCl, 1 MgCl_2_, 1.25 KH_2_PO_4_, 26 NaHCO_3_, 1 sodium pyruvate, and 10 glucose. Transverse coronal brain slices (200 µm) were prepared using a Vibratome (Leica, VT100S). Slices were equilibrated with an oxygenated artificial cerebrospinal fluid (aCSF) for >1 h at 32°C before transfer to the recording chamber. The slices were continuously superfused with aCSF at a rate of 1.5 ml/min containing the following (in mM): 113 NaCl, 3 KCl, 1 NaH_2_PO_4_, 26 NaHCO_3_, 2.5 CaCl_2_, 1 MgCl_2_, and 5 glucose in 95% O_2_/5% CO_2_.

### Electrophysiological Recordings

Brain slices were placed on the stage of an upright, infrared-differential interference contrast microscope (Olympus BX50WI) mounted on a Gibraltar X-Y table (Burleigh) and visualized with a 40X water-immersion objective by infrared microscopy (Olympus OLY-150). Cholinergic neurons were identified by the presence of enhanced green fluorescent protein (eGFP) resulting from expression of the Chat- *tau*GFP transgene. The internal solution for voltage clamp experiments contained (in mM): 130 KCl, 5 CaCl_2_, 10 EGTA, 10 HEPES, 2 MgATP, 0.5 Na_2_GTP, and 10 phosphocreatine, for current clamp experiments (in mM): 115 K-Gluconate, 10 KCl, 10 HEPES, 10 EGTA, 0.5 Na_2_GTP, 2 MgATP and 10 phosphocreatine. All recordings were conducted at 28°C. GABAergic currents were isolated with the addition of 6-Cyano-7-nitroquinoxaline-2,3-dione (CNQX, 10 µM; Abcam) and D-amino-phosphovaleric acid (D-AP-5, 50 µM; Abcam) and glutamatergic currents were recorded in the presence of bicuculline (10 µM; Abcam). Membrane currents were recorded with a Multiclamp 700B or an Axopatch 200B (Molecular Devices) in whole-cell configuration. Electrophysiological signals were low-pass filtered at 2–5 kHz, stored on a PC and analyzed offline with pClamp 10 software (Molecular devices).

### Analysis of Spontaneous Miniature IPSCs

Spontaneous miniature inhibitory and excitatory postsynaptic currents were recorded in the presence of tetrodotoxin (TTX) (1 µM; Sigma-aldrich). Autodetected events with a scaled template were also visually examined to correct for noise fluctuation (Clampfit 10, Molecular devices). Analysis of mini IPSC decay phase (Clampfit 10, Molecular Devices) was based on the following criteria: (1) single events only (i.e., no multiple events), (2) events having stable baselines 15 ms before the rise, and (3) smooth transition from 0 current to peak amplitude [<20% deviation in *d*(pA)/*dt*) during rise]. Aligned GABAergic synaptic currents were averaged and the decays were fit by a double-exponential function. Glutamatergic synaptic currents were fit by a single-exponential function.

### Immunocytochemistry

FOS staining was prepared from Chat -*tau*GFP mice at postnatal age 2 months ±1 week. Animals were deeply anaesthetized with a mixture of Ketamine and Xylazine and perfused transcardially with 10 ml of cold 1xPBS followed by 25 ml of 4% paraformaldehyde (PFA). Brains were immediately removed, postfixed in 4% PFA at 4°C overnight, cryoprotected in a 30% (w/w) sucrose solution at 4°C for 3 days, frozen in Tissue-Tek (Sakura), and stored at −80°C. The complete rostral to caudal extension of the DMH from Bregma level −1.5 to −2.4 was cut in 30 µm coronal sections with a Cryostat. Sections were mounted on slides, washed with 1xPBS, permeabilized with 0.2% Triton X-100 and blocked with 5% normal donkey serum for 2 h at room temperature. Slides were then incubated in primary rabbit antibody to c-fos (1∶750, Abcam, ab7963) for 48 h at 4°C, followed by washing and 1 h of incubation with an Alexa-594 conjugated secondary donkey antibody (1∶500) at room temperature. Labeled probes were cover slipped with DAPI-Fluoromount-G (Southern Biotech). Positive controls for the c-fos protocol were provided by examination of regions of high neuronal activity (e.g. olfactory bulb). Images were acquired using the Olympus Spinning Disk Confocal microscope (DSU; Olympus). The DAPI signal was used to confirm that immunodetection of c-fos was exclusively present in cell nuclei. Chat+ neurons of complete 30 µm sectioned DMH sets were counted as expressing positive c-fos immunoreactivity. The observer was blind to the experimental condition of each individual mouse.

Biocytin filling was performed by recording from Chat+ neurons in the DMH with a K-Gluconate intracellular solution as described above, additionally containing 0.4% (w/w) Biocytin. Slices were processed with standard staining procedures. In brief, following the termination of recordings the 200 µm thick slices were submerged in 4% PFA/1xPBS for 1 week at 4°C, washed in 1xPBS, permeabilized with 1% Triton for 30 minutes at room temperature, and then incubated with Alexa 594 conjugated streptdavidin (1∶1000, Molecular Probes) for 4 h at room temperature. After washing labeled probes were cover-slipped with Fluoromount G (Southern Biotech) and images acquired with the Olympus Spinning Disk Confocal microscope (DSU; Olympus).

### Statistics

Statistical analyses were performed on data obtained from Chat-positive neurons using the independent t-test. The mean values were reported from the entire population tested (Origin 8.0). Data were considered significantly different when the P value was <0.05. All statistical results are given as means ± S.E.M.

## Results

### Expression of Cholinergic Neurons in the DMH

**Figure 1:**
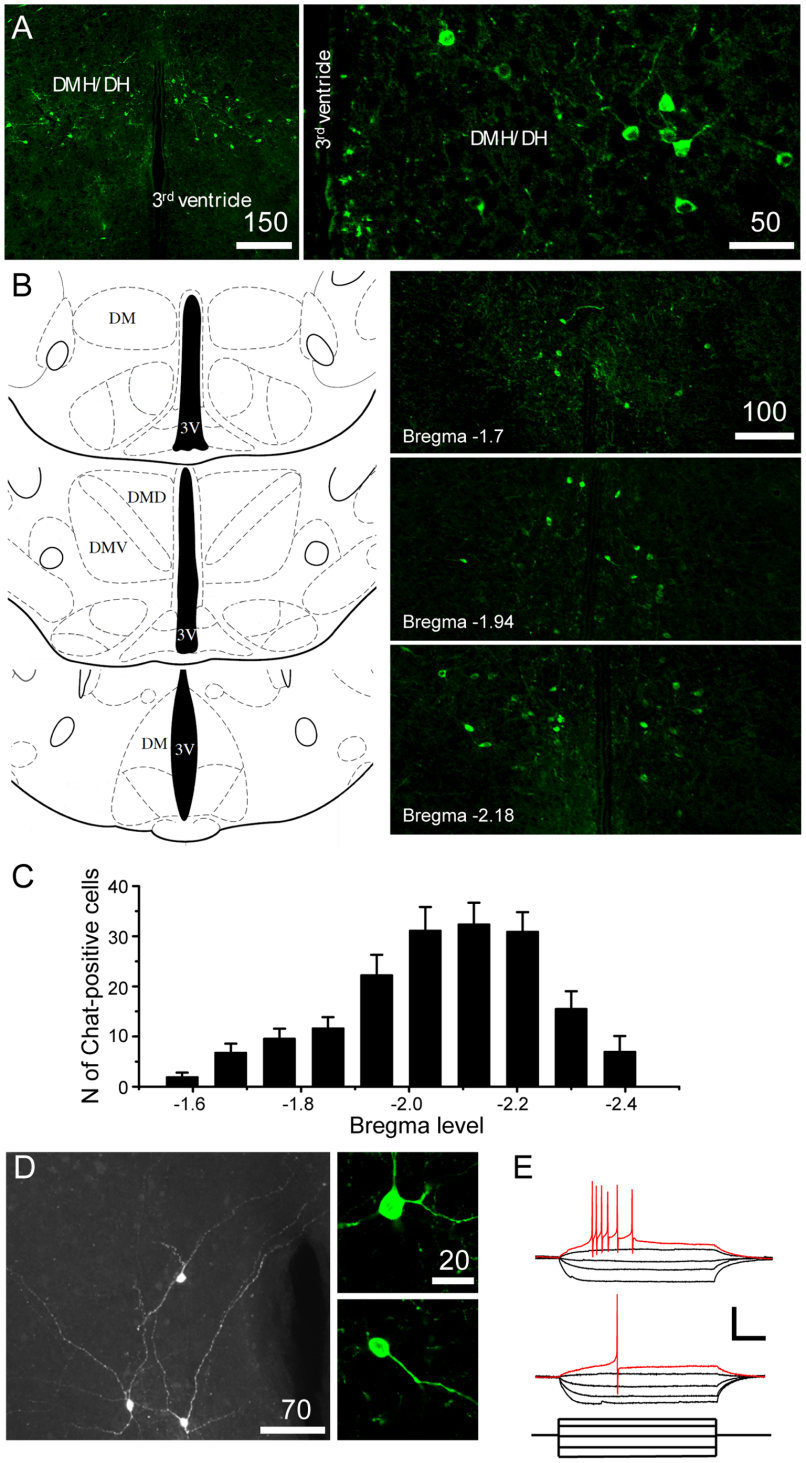
Cholinergic neurons in the DMH. A. Images of fluorescence microscopy showing the expression of Chat-positive neurons (green) in the DMH of Chat-*tau*GFP mice. The distribution of cholinergic neurons within the hypothalamus was restricted to the DMH. B. Image of fluorescence microscopy showing the distribution of Chat-positive neurons (green) at three different levels from Bregma (Bregma −1.7, −1.94 and −2.18; Right panel). Left panel: The reference diagrams were adapted from the Mouse Brain Atlas of Paxinos and Franklin (2nd edition, 2001). C. Graph of the number of Chat-positive neurons at the different levels from Bregma. D. Morphology of Chat-positive neurons. Left panel: Immunocytochemical staining combined biocytin labeling of Chat-positive cells. There were two major Chat+ cell types. Right panel: image of fluorescence microscopy of GFP-expressing neurons (upper panel: multipolar-shaped cell, bottom panel: oval or bipolar-shaped cell). E. Responses of Chat-positive neurons to hyperpolarizing and depolarizing current steps. Type I showed a burst of action potentials (upper panel), whereas Type II fired only a single action potential in response to a sustained depolarizing current injection. Scale bar: 50 mV, 100 pA and 100 ms.

DMH neurons project to the arcuate nucleus, which contains NPY and POMC-expressing neurons. Arcuate NPY and POMC neurons are innervated by cholinergic axons [18], consistent with the idea that hypothalamic cholinergic neurons regulate the excitability of orexigenic and anorexigenic neurons in the arcuate nucleus, thereby modulating feeding behavior. We first examined whether cholinergic neurons are detected within the DMH using a transgenic animal model that selectively expresses eGFP in Chat-positive neurons [22]. We found that a subpopulation of DMH neurons were Chat-positive neurons (Fig. 1A). These Chat-positive neurons were located from the rostral to caudal boundaries of the DMH (Fig. 1B and C), with a relatively higher expression of Chat-positive neurons in the mid and caudal portions of the DMH (Bregma −2.1 mm; Fig. 1B and C). The overall number of cholinergic neurons in the DMH was 168.6±16.6 (n = 15 animals).

Chat-positive neurons were classified into two major groups on the basis of their morphology; one group included large multipolar – shaped cells with, at least, three projections emitting from the cell body. The other included small round and/or oval-shaped Chat-positive neurons (Fig. 1D). Immunocytochemical staining combined with biocytin labeling of Chat-positive cells confirmed two predominant morphological classes of cholinergic neurons in the DMH (Fig. 1D). Some of the cholinergic neurons appeared to be in physical contact with one another (Fig. 1D) and to send projections to the dorsal and lateral hypothalamus, while other projections extended to the contralateral part of the hypothalamus (Fig. 1D).

Our electrophysiological analysis also indicated that there were two distinct types of Chat-positive neurons based on their intrinsic properties. The first type of Chat-positive neuron (Type I) displayed a burst of 3–5 action potentials in response to a depolarizing current injection (n = 10 out of 35 neurons), whereas the other type responded to depolarization with a single spike (Type II; n = 25 out of 35 neurons; Fig. 1E). These observations are consistent with the findings of Crosby and colleagues [14] showing that there are two distinct populations of DMH neurons. However, the mean membrane resistance (Type I: 694.3±55.1 MΩ, Type II: 809.2±40.8 MΩ; n = 8 neurons and n = 35 neurons, respectively; p>0.05) as well as the average maximum frequency of spikes (Type I: 70±10 Hz vs. Type II: 73±4 Hz at 79 pA injection; n = 10 neurons and n = 25 neurons, respectively; p>0.05) were not significantly different. Furthermore, there was no correlation between the morphology and the intrinsic property of the two types of Chat-positive neurons.

### Overnight Fasting Increases Fos Expression in Chat-positive Neurons

**Figure 2:**
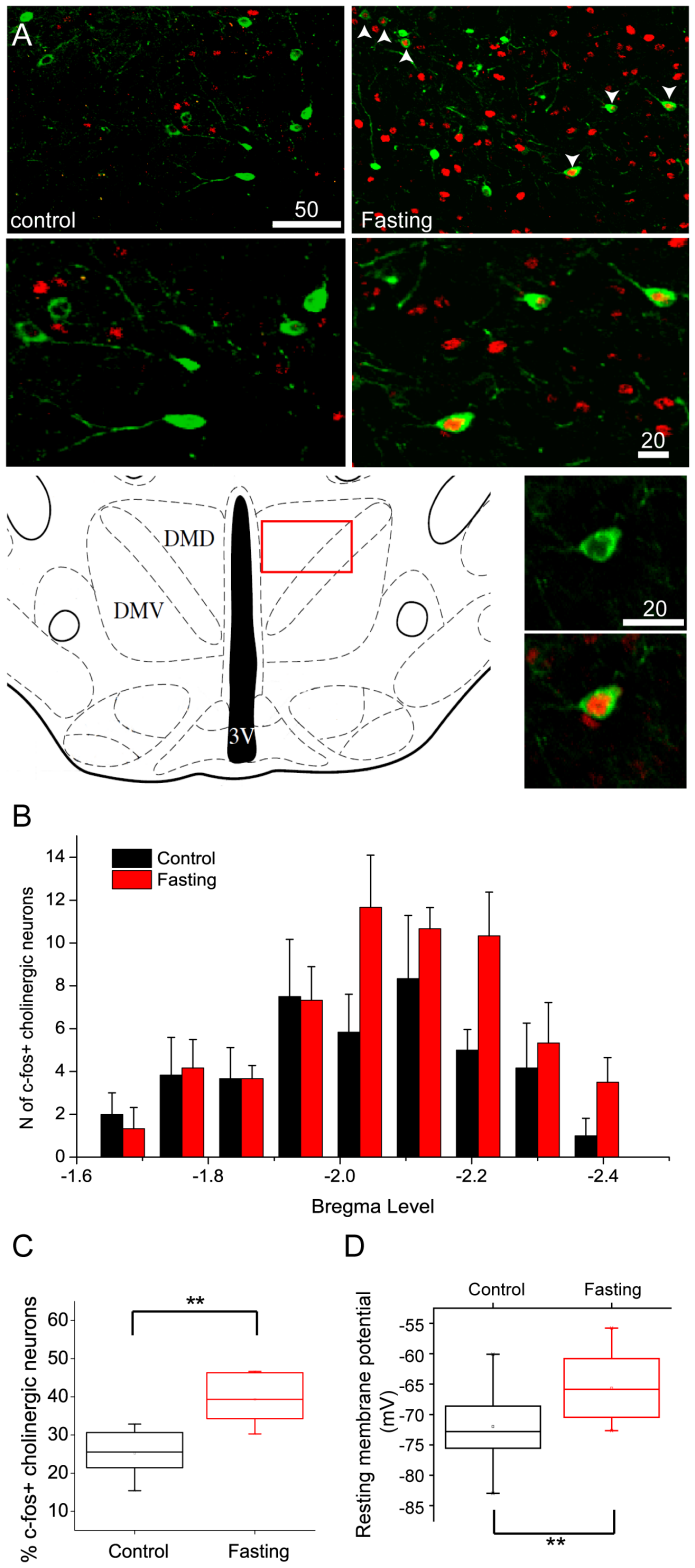
Overnight food deprivation increases the expression of fos protein and shifts the resting membrane potential of DMH cholinergic neurons. A. C-fos expression in the cholinergic neurons in the DMH from control (upper left panel) and fasting groups. Fasting induced c-fos expression in the DMH (upper right panel: white arrowhead and middle right panel). The reference diagram shows the area where cholinergic neurons were observed (bottom left panel). Bottom right panels are higher magnification views of the c-fos expression in cholinergic neurons under control (top) and following overnight fasting (bottom) conditions. B. Rostral to caudal distribution of c-fos expression in cholinergic neurons following overnight food deprivation. Control: black bars (n = 6 animals); Overnight fasting: red bars (n = 6 animals). C. Box plot of the fraction of cholinergic neurons showing c-fos expression in control (black) and fasting (red) conditions. Fasting significantly increased the expression of c-fos protein in cholinergic neurons. D. Box plot of resting membrane potentials measured in cholinergic neurons under control and post fasting conditions. Overnight fasting resulted in a significant shift in the resting membrane potential to more depolarized levels (Control: n = 15 neurons, Fasting: n = 11 neurons. p<0.05).

Although DMH neurons are implicated in ingestive behavior [9], there is little information about the phenotypes of DMH neurons that are responsible for the regulation of food intake. Thus, we performed c-fos immunocytochemistry following overnight food deprivation to determine whether Chat-positive neurons in the DMH are altered in their activity profile in response to the availability of nutrients. We found that overnight fasting induced the expression of c-fos protein in Chat-positive neurons and other cells in the DMH (Fig. 2A and B). The percentage of c-fos-positive cholinergic neurons was significantly increased from 25.2±2.7% to 39.4±2.8% (Fig. 2C; control: n = 6 animals vs. Fasting: n = 6 animals; p<0.01). We found that c-fos-positive cholinergic cells following overnight fasting were observed from the rostral to caudal boundaries of the DMH of the hypothalamus (Fig. 2B). Consistent with the idea that the expression of c-fos protein reflects increased neuronal activity, the mean resting membrane potentials of Chat-positive neurons from control and fasting groups were significantly different. Overnight food deprivation elevated the mean resting membrane potential from −72.4±1.6 mV to −65.7±1.6 mV (n = 15 neurons, vs. n = 11 neurons; p<0.05; Fig. 2D). Both immunocytochemical and electrophysiological data thus indicate that overnight food deprivation increases the activity of Chat-positive neurons in the DMH, which in turn induces c-fos expression. Our data are similar to the findings of increased c-fos expression in the DMH from animals in restricted feeding contexts [23].

### Overnight Fasting Decreases Spontaneous GABAergic Synaptic Activity

**Figure 3:**
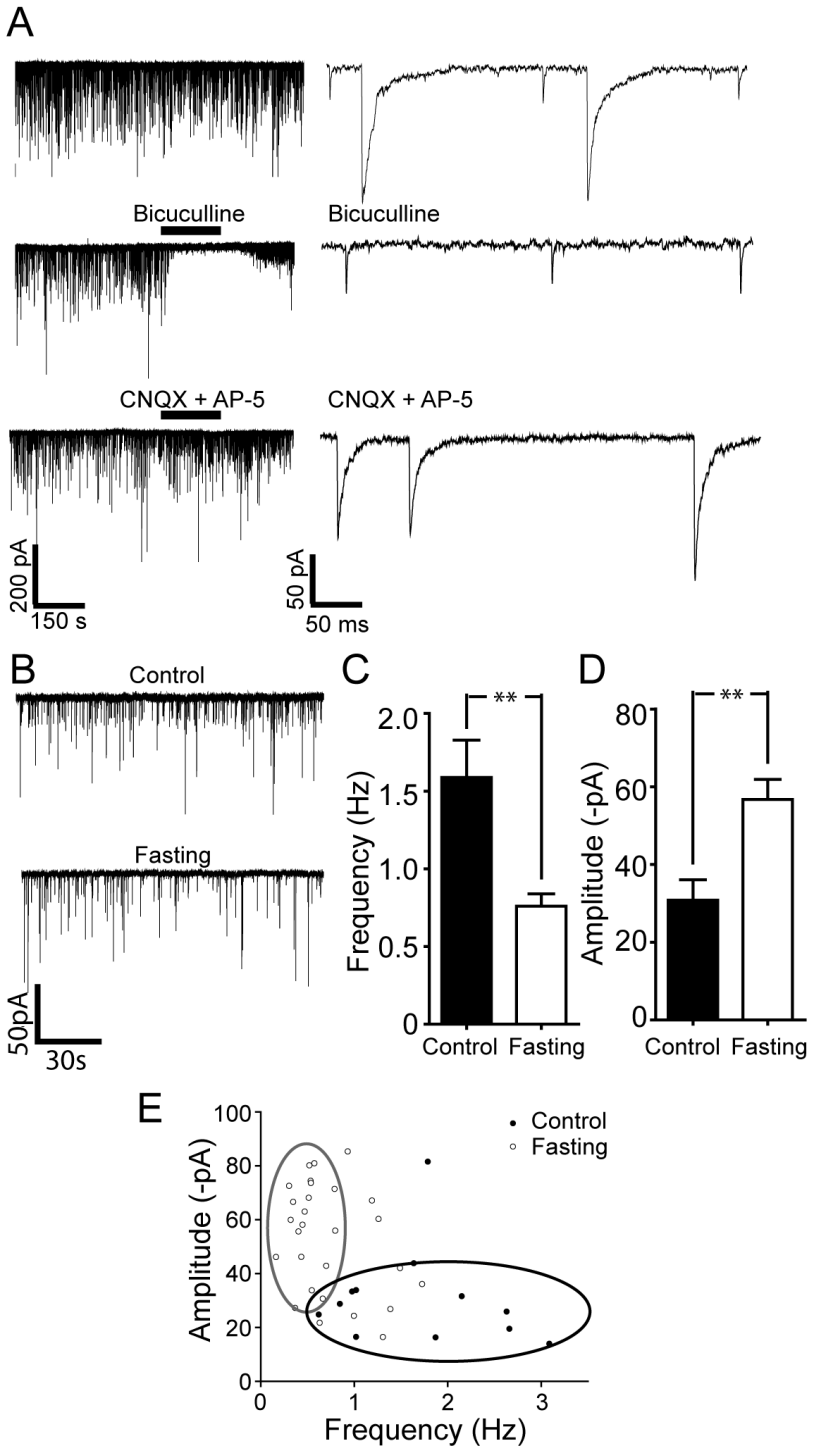
Overnight food deprivation alters the profile of synaptic inputs to DMH cholinergic neurons. A. Representative recordings of sPSCs activity in identified cholinergic neurons. Two components of PSCs were evident at a holding potential (HP) of −70 mV (upper right panel: on the expanded time scale). Middle panel shows that the GABA_A_ receptor antagonist, bicuculline completely blocked slow synaptic currents (middle panel; right: expanded time scale), while the glutamate receptor antagonists abolished fast synaptic currents (bottom panel, right: expanded time scale). Thus, the DMH cholinergic neurons received both GABAergic and glutamatergic currents. B. Synaptic activity in DMH cholinergic neurons under control and post-fasting conditions. Sample traces show typical examples of the spontaneous synaptic currents recorded in cholinergic neurons under control vs. overnight fasting conditions. C and D. Pooled data of the frequency (C) versus the amplitude (D) of sPSCs from 13 and 29 DMH cholinergic neurons in control and fasting groups. Overnight fasting significantly modulated the frequency as well as the amplitude of sPSCs. E. Plot of amplitude vs. frequency values for sPSCs under control vs. overnight fasting conditions.

We next examined whether short-term food deprivation alters excitatory and/or inhibitory tone to Chat-positive cells in the DMH. As shown in Fig. 3A, Chat-positive cells received both fast and slow synaptic currents: the fast components were blocked by the glutamate receptor antagonists, CNQX and AP-5, whereas the slow components were abolished by the GABAA receptor antagonist, bicuculline. These data are consistent with the conclusion that cholinergic neurons were innervated by both GABAergic and glutamatergic neurons. We found that overnight food deprivation significantly changed the overall baseline synaptic activity (Fig. 3B, C and D). The mean frequency of spontaneous postsynaptic currents (sPSCs) was changed from 1.6±0.2 Hz (control) to 0.8±0.1 Hz (Fig. 3C and E; n = 13 neurons and n = 29 neurons; p<0.01) and the mean amplitude of sPSCs from 30.8±5.3 pA (control) to 56.8±5.2 pA (Fig. 3D and E; n = 13 neurons and n = 29 neurons; p<0.01).

**Figure 4:**
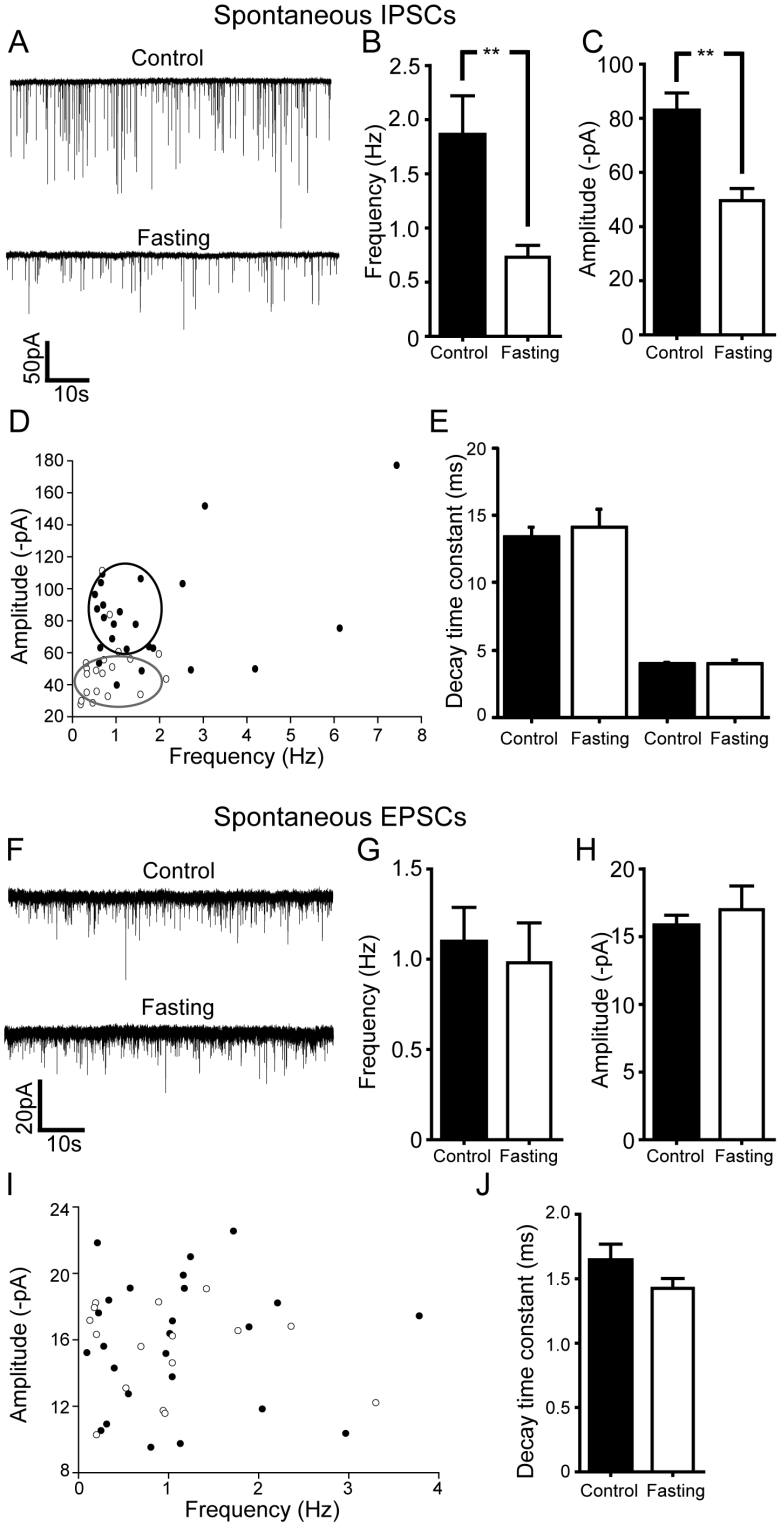
Overnight food deprivation results in significant reductions in net inhibitory tone to DMH cholinergic neurons. A. Spontaneous GABAergic (IPSC) activity in DMH cholinergic neurons under control vs overnight fasting conditions. There was a significant decrease in the GABAergic transmission following overnight fasting. B and C. Summary plots of the changes in frequency (control: n = 24 neurons, Fasting: n = 19 neurons) and amplitude (control: n = 24 neurons, Fasting: n = 19 neurons) of GABAergic sIPSCs in DMH cholinergic neurons following overnight fasting conditions. Both parameters were significantly decreased. D. Pooled data showing the frequency versus the amplitude of sIPSCs. Food deprivation decreased both the frequency and the amplitude of sIPSCs recorded in DMH cholinergic neurons (Filled circle, control; open circle, fasting). E. Graph showing no significant difference in the decay time constant of sIPSCs in the DMH cholinergic neurons under control and fasting conditions. F. Sample recording traces of pharmacologically isolated glutamatergic s EPSCs in control vs. post-fasting conditions. G and H. Summary plots showing the frequency and the amplitude of sEPSCs in control and fasting groups. There was no change in both parameters. I. Pooled data comparing the frequency vs. the amplitude of sEPSCs in DMH cholinergic neurons under control (filled circle) vs. post fasting (open circle) conditions. J. Graph shows no difference in the decay time constant of sEPSCs in control and fasting groups.

As there was a significant difference between these parameters, we determined whether overnight fasting alters inhibitory and/or excitatory synaptic input to cholinergic neurons. We found that overnight food deprivation strongly attenuated inhibitory tone to Chat-positive neurons (Fig. 4A, B, C and D). With CNQX (10 µM) and DL-APV (50 µM) to isolate GABAergic postsynaptic currents (sIPSCs), we found a significant reduction of GABAergic synaptic tone onto cholinergic neurons in fasting group. The mean frequency of sIPSCs was decreased from 1.9±0.4 Hz to 0.7±0.1 Hz following overnight fasting (Fig.4B and D; n = 24 neurons vs. n = 19 neurons; p<0.01). This was associated with decreased mean amplitude of sIPSCs from 82.7±6.6 pA to 49.6±4.5 pA. (Fig. 4C: n = 24 neurons vs. 20 neurons; p<0.01). There was no change in the decay time constant of sIPSCs, consistent with a lack of change in post synaptic GABAergic receptors (Fig. 4E; control: τ_fast_: 4.0±0.1 ms, τ_slow_: 13.4±0.7 ms, fasting: τ_fast_: 4.0±0.3 ms, τ_slow_: 14.1±1.4 ms; n = 28 neurons and n = 18 neurons, respectively).

In the presence of bicuculline (10 µM) to block GABAergic currents, overnight fasting had no effect on any of the parameters of glutamatergic synaptic transmission (sEPSCs; Fig. 4 F-J). Thus, neither the frequency, amplitude nor decay time constant of sEPSCs were altered in control and overnight fasting conditions (Fig. 4H, I, J and K; Frequency: control: 1.1±0.2 Hz vs. fasting: 1.0±0.2 Hz; Amplitude: control: 15.8±0.8 pA vs. fasting: 17.0±1.8 pA; Decay time constant: control: 1.6±0.1 ms vs. fasting: 1.4±0.1 ms; p>0.05; n = 25 neurons and 16 neurons, respectively).

Taken together these data indicate that the decreased inhibitory tone may lead to a net increase in the mean resting membrane potential and increased activity of Chat-positive neurons in the DMH following overnight food deprivation. These parameters are likely to underlie the observed induction of c-fos expression in cholinergic neurons.

### Overnight Fasting Alters Inhibitory Tone to Cholinergic Neurons via a TTX Sensitive Mechanism

**Figure 5:**
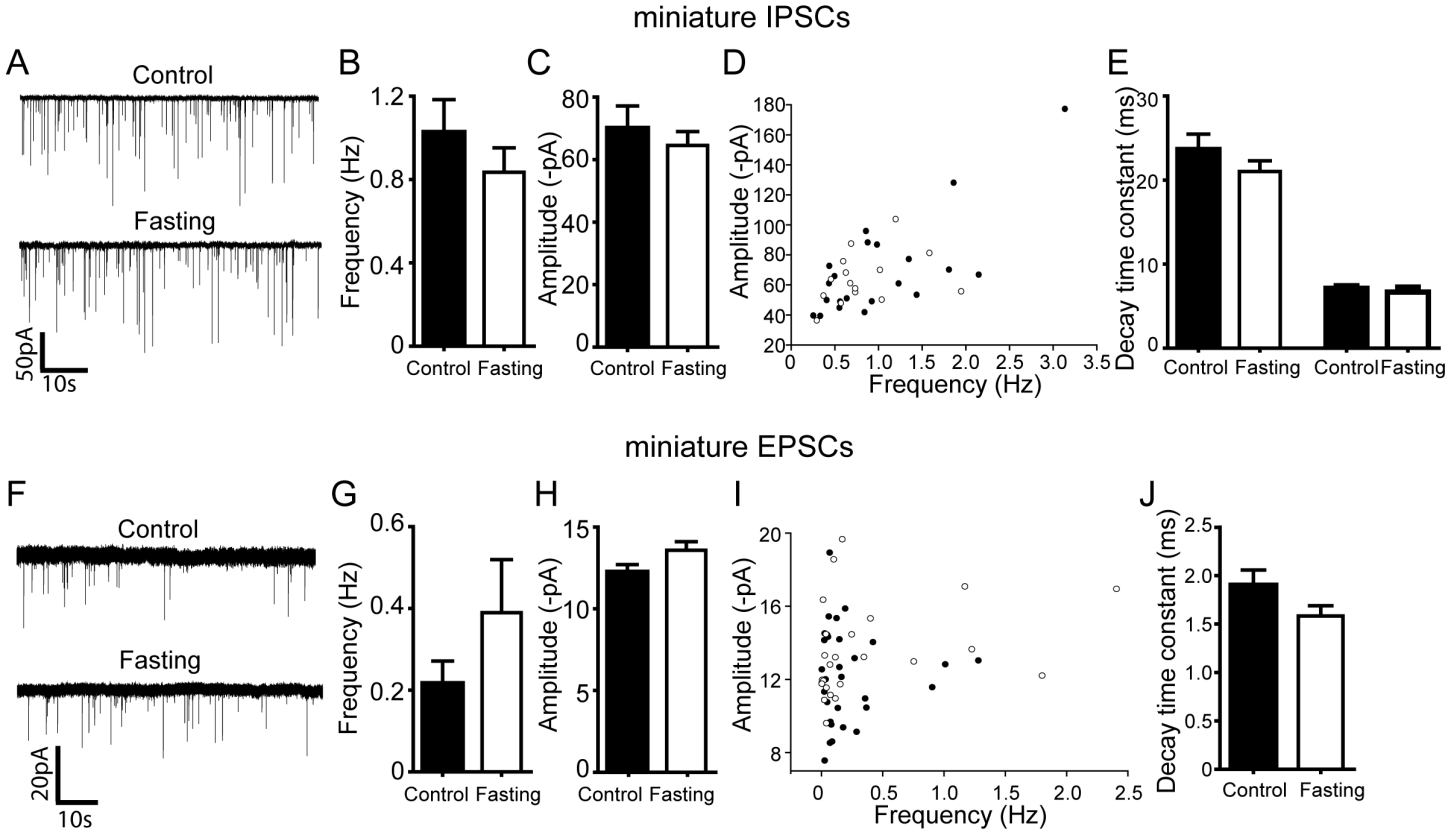
Overnight food deprivation does not alter TTX resistant inhibitory or excitatory synaptic currents recorded in DMH cholinergic neurons. A. Examples of electrophysiological recordings showing miniature (TTX resistant) GABAergic synaptic currents in DMH cholinergic neurons following control vs. overnight fasting conditions B and C. Summary plots show that fasting did not alter miniature GABAergic synaptic transmission (control: n = 21 neurons, Fasting: n = 15 neurons). D. Pooled data showing no difference in the frequency or amplitude of miniature IPSCS recorded in DMH cholinergic neurons (Filled circle, control; open circle, fasting). E. There was no change in the decay time constants of miniature IPSCs recorded in DMH cholinergic neurons. F. Sample recording traces showing miniature glutamatergic synaptic transmission (mEPSCs). G, H, I and J. Pooled data show that overnight fasting did not change the frequency, amplitude and decay time constant of miniature EPSCs (n = 31 neurons, Fasting: n = 24 neurons; Filled circle, control; open circle, fasting; control).

We further investigated the mechanism by which overnight fasting decreases inhibitory tone by including the action potential inhibitor - TTX - in the aCSF solution to block neuronal network activity. Under these experimental conditions, we found that overnight fasting was without effect on all parameters tested. Thus, neither the frequency nor amplitude of the miniature IPSCs differed between the control and overnight fasting (Fig. 5A-E; Frequency: Control: 1.0±0.2 Hz vs. Fasting: 0.8±0.1 Hz; Amplitude: Control: 70.0±7.2 pA vs. Fasting: 64.5±4.5 pA; Control: τ_fast_: 7.1±0.3 ms, τ_slow_: 23.6±1.8 ms, Fasting: τ_fast_: 6.7±0.6 ms, τ_slow_: 21.0±1.3 ms; p>0.05; n = 21 neurons and n = 15 neurons, respectively). Likewise, overnight fasting had no effect on any parameters of miniature EPSCs. (Fig. 5F-J; Frequency: Control: 0.2±0.1 Hz vs. Fasting: 0.4±0.1 Hz; Amplitude: Control: 12.2±0.5 pA vs. Fasting: 13.6±0.5 pA; Decay time constant: Control: 1.9±0.2 ms vs. Fasting: 1.6±0.1 ms; p>0.05; n = 31 neurons and 24 neurons, respectively). These data indicate that the availability of nutrients regulates superthreshold activation of GABAergic inputs to cholinergic DMH neurons, thereby indirectly controlling their excitability.

## Discussion

The current study provides physiological evidence that a subpopulation of DMH neurons are cholinergic and that GABAergic inhibitory inputs to these cholinergic neurons are modulated by the availability of nutrients. Overnight food deprivation induced c-fos expression in cholinergic neurons in the DMH, which was associated with an increased baseline resting membrane potential. In addition, GABAergic, but not glutamatergic, synaptic transmission to cholinergic neurons was significantly diminished following overnight food deprivation. The increased excitability of DMH cholinergic neurons was dependent on neural network activity since TTX completely abolished the observed synaptic changes. Based on these data, we suggest that cholinergic neurons in the DMH readily detect and respond to changes in nutrient status through GABAergic presynaptic inputs. We propose that reduced inhibitory tone and the consequent enhancement of excitability of cholinergic neurons may be an important factor driving feeding in mice.

Central cholinergic neurons are found gathered together in nuclei such as in the basal forebrain and brainstem regions, or dispersed as interneurons throughout brain areas such as the striatum [24]. The distribution of cholinergic neurons in the hypothalamus is rather scattered [24], [25]. The Chat-positive cells in the DMH are small- and medium-sized bipolar cells or relatively large triangular shaped cells [25], consistent with our findings that cholinergic neurons were subdivided into two populations on the basis of their morphology. In addition, a cluster of Chat-positive neurons are found in the DMH and DH [20], [24]. Using transgenic Chat-*tau*GFP mice [22], we found that a subset of the DMH neurons were indeed Chat-positive cells but that the DMH cholinergic neurons within the DMH totaled only ∼200 cells/animal, in contrast with the relative abundance of neuropeptide-expressing neurons in the hypothalamus, such as POMC neurons (∼3000 neurons) [26], [27]. Importantly, we noted that the Chat positive neurons were strictly confined to the DMH and/or DH. Hence, cholinergic neurons can be used as a nucleus-specific, albeit low abundance, marker for the DMH.

There is considerable evidence demonstrating that the DMH plays an important regulatory role in the maintenance of overall energy balance. For example, lesions of the DMH induce anorexigenic effects (see for review [9]). Deletion of brain-derived neurotrophic factor (BDNF) in the DMH results in hyperphagic behaviors [13] and knock-down of NPY expression in the DMH reduces body adiposity and food intake, but enhances thermogenesis by promoting the development of brown adipocytes [5]. A recent study further shows that DMH neurons co-release retrograde signaling molecules, such as endocannabinoids and nitric oxide, that are regulated by fasting [28]–[30]. Thus a subpopulation of DMH neurons, perhaps including the DMH cholinergic neurons, appears to participate in the control of ingestive behavior.

It is well known that nicotine acts as a strong anorexigenic substance [16]. A recent elegant study by Picciotto and colleagues shows the cellular mechanisms that underlie the anorexigenic effects of nicotine [17]. Nicotine activates α3β4-containing nicotinic receptors on POMC neurons and thereby, reduces energy intake. In line with these findings, POMC neurons in the arcuate are innervated by cholinergic inputs [18], which suggests that not only nicotine but its endogenous counterpart, acetylcholine, affects feeding behavior via the melanocortinergic system. Interestingly, the activation of muscarinic receptors, in particular type 3, induces opposing effects on feeding behavior. For instance, M3-deficient mice exhibit decreased food intake as well as body weight [19]. Furthermore, deletion of the M3 muscarinic receptor alters the POMC: AgRP ratio and interferes with the ability of AgRP to stimulate food intake [19]. Thus the relative activation of nicotinic vs. muscarinic cholinergic receptors in the hypothalamus may be an important determinant of the net output of melanocortinergic signaling to second order neurons. It has not been clearly defined whether cholinergic inputs onto melanocortinergic neurons or other hypothalamic neurons are originated solely from the brain stem, including the pedunculopontine and laterodorsal tegmental nuclei. As the DMH contains cholinergic neurons, these cholinergic neurons would send projections to hypothalamic nuclei such as the arcuate, PVN and LH. Indeed these areas have been shown to be innervated by DMH neurons [21].

As both nicotinic and muscarinic receptors influence ingestive behavior, the regulation of cholinergic neuronal activity would be a critical factor determining orexigenic vs. anorexigenic effects of acetylcholine. In other words, levels of acetylcholine at hypothalamic synapses differentially activate nicotinic vs. muscarinic receptors, thereby oppositely modulating food intake. In our current study, we found that only 12 hours of food deprivation was sufficient to dramatically reduce inhibitory tone to cholinergic neurons, which resulted in increased excitability of cholinergic neurons. Likewise, food deprivation induced c-fos expression in DMH cholinergic neurons. Hence DMH cholinergic neurons are able to sense the availability of nutrients mainly via presynaptic GABAergic inputs after only 12 hours of food deprivation. However, prolonged food deprivation and/or long-term dietary-restriction may differentially influence cholinergic neuronal activity. For instance, the DMH neurons co-release retrograde signal molecules, including endocannabinoids and NO, which in turn regulate GABAergic input in an opposite manner [14]. Acute food deprivation for 24 hours strengthens GABAergic tone via downregulation of presynaptic cannabinoid type 1 receptors [14]. It has also been shown that 24 hrs fasting reduces neuronal nitric oxide synthase mRNA expression in the DMH as well as the medial preoptic area [28]. Since NO could induce GABAergic LTP at synapses onto DMH neurons, altered production of NO would affect GABAergic synaptic plasticity. Importantly, DMH neurons receive GABAergic inputs mainly from the preoptic area [7], [8], [10], [31] and these inhibitory inputs appear to be important in regulating thermogenesis. In addition, activation of melanocortin receptor type 4 selectively expressed in cholinergic neurons lowers body weight, improves energy expenditure and reduces hyperglycemia and hyperinsulinemia [32].The cholinergic neurons in the DMH could play a critical role in controlling not only energy intake but also energy expenditure. Thus, the extent of disinhibition of cholinergic neurons may determine the degree of output of acetylcholine and, perhaps, the ratio of nicotinic vs. muscarinic receptor-mediated outputs. Such a subtle tuning of hypothalamic cholinergic signaling will act as a gate that controls metabolic signals between the brain and target areas.

### Conclusion

Both nicotinic and muscarinic cholinergic signaling pathways have been implicated in ingestive behavior [16], [17], [19]. Hypothalamic cholinergic neurons are found in the DMH, which receives information on environmental temperature as well as nutrient status [9], [10] and relays this information to other hypothalamic nuclei, including the PVN, LH, SCN, and ARC [33], [34]. The activity of DMH cholinergic neurons appears to be strongly regulated by GABAergic inhibitory tone from the median preoptic area [7] and the DMH neurons, possibly including cholinergic neurons, regulate the strength of inhibitory tone via feedback mechanisms using retrograde signaling molecules [14]. Our data support the idea that synaptic plasticity at synapses onto DMH cholinergic neurons may contribute to the control of overall ingestive behavior. Additional studies are necessary to specifically address the physiological importance of hypothalamic cholinergic neurons for whole-body energy balance and the development of obesity.
